# Adult‐onset neuronal intranuclear inclusion disease presenting with typical MRI changes

**DOI:** 10.1002/brb3.1477

**Published:** 2019-11-20

**Authors:** Xinsheng Han, Miao Han, Ning Liu, Jianke Xu, Yan Zhang, Yun Zhang, Daojun Hong, Wei Zhang

**Affiliations:** ^1^ Department of Neurology Kaifeng Central Hospital Kaifeng China; ^2^ Department of Neurology Peking University People's Hospital Beijing China; ^3^ Department of Neurology Peking University First Hospital Beijing China

**Keywords:** acute encephalopathy syndrome, magnetic resonance imaging, neuronal intranuclear inclusion disease

## Abstract

**Background:**

This study aims to analyze the clinical, imaging, electrophysiological, and dermatopathological features of a patient with adult‐onset neuronal intranuclear inclusion disease (NIID) and to explore the diagnostic methods of adult‐onset NIID.

**Case presentation:**

We here report a 63‐year‐old male with recurrent acute encephalopathy syndrome and autonomic nervous system damage syndrome characterized by sexual dysfunction and urinary and fecal dysfunction. Cranial diffusion‐weighted magnetic resonance imaging (DWI) demonstrated symmetrically distributed strip‐shaped high‐intensity signal in bilateral fronto‐occipital‐parietal cortical‐medullary junction. Electrophysiological test revealed that the main site of injury was myelin sheath in both motor and sensory nerves. Skin biopsy revealed eosinophilic spherical inclusion bodies in the nucleus of sweat gland epithelial cells.

**Conclusion:**

This case suggests that adult NIID is a chronic neurodegenerative disease with high clinical heterogeneity. Subcortical strip‐shaped high‐intensity signal on DWI has high diagnostic significance. Eosinophilic intranuclear inclusion bodies detected by skin biopsy contribute to diagnosis.

## Graphical abstract

Adult‐onset neuronal intranuclear inclusion disease (NIID) has a low incidence, diverse clinical manifestations, and difficulty in early diagnosis. NIID has typical clinical manifestations and suggestive MRI features. The use of skin biopsy with minimal trauma can promptly diagnose NIID and improve the awareness of the clinicians.

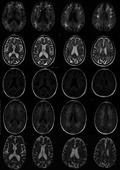

## 1 INTRODUCTION

Neuronal intranuclear inclusion disease (NIID), first proposed by Lindenberg in 1968, is a rare disease based on the appearance of intranuclear inclusions in cells of the brain and visceral organ (Lindenberg, Rubinstein, Herman, & Haydon, 1968). The pathogenesis of NIID is still unclear. Clinically, NIID is characterized by multiple systemic damage syndromes mainly in the central and peripheral nervous systems. In recent years, NIID can be diagnosed by skin biopsy and characteristic findings on magnetic resonance imaging. Consequently, the number of NIID cases that reported has increased (Abe & Fujita, 2017; Kawarabayashi et al., 2018; Motoki et al., 2018). We report an adult‐onset NIID patient with typical clinical manifestations, characteristic MRI findings, and pathological changes.

## 2 CASE PRESENTATION

The patient, a 63‐year‐old male, of Han nationality and right‐handed, was admitted to the hospital with the presence of “recurrent headache, personality change, and abnormal mental behavior for 3 years.” Three years ago, the patient developed a headache without obvious incentives, which was manifested as headache, irritability, bad‐tempered, paranoia without fever, limb twitching, or conscious disturbance. The above symptoms recurred repeatedly, lasting from several hours to several days each time. The frequency of attack was not constant, sometimes once or more than 10 times in a month and sometimes once in a few months. The symptoms could be restored to normal level during the interictal period. The patient had erectile dysfunction for 13 years, and he experienced dysuria, constipation, and sweating in his hands. He also had 6 years of hypertension, 4 years of blindness in the left eye, 1 year of cataract in the right eye, 30 years of smoking (20 cigarettes a day), and 30 years of drinking (250 g a day). He has one son and one daughter, and his daughter is in good health. His son was bad‐tempered, introverted, and committed suicide at the age of 20 years. His son had not experienced significant fluctuating psychosocial disorders before his death. The other members of the family do not have a similar medical history.

One day before admission, the patient had a headache again, mainly in the forehead, with eyeball swelling and pain, nausea, and vomiting. Vomit was noncoffee gastric contents. Seven hours before admission, the patient appeared unconscious, restless, and gibberish, did not wear clothes, and did not know where he was. The patient complained of seeing nothing, and his symptoms were fluctuating, then he was admitted to the hospital.

Physical examination revealed temperature of 36.5°C, pulse of 97 times/min, respiration of 20 times/min, and blood pressure of 158/104 mmHg. The patient's consciousness was ambiguous, euphoric, and irritable. The patient could not cooperate with the measurement of the orientation to person, place, and time; he can understand and execute simple instructions, and cannot cooperate in the higher cortical function or binocular visual acuity tests. The patient had fluent speech. His bilateral frontal lines and nasolabial sulcus were symmetrical. The tooth angle was not skewed, and the tongue was centered. The limbs had normal muscle strength and moderate muscle tension. Tendon reflex of extremities was sluggish (+), and the bilateral Babinski and Chaddock signs were positive bilaterally. The patient was not cooperated in the sensory system and ataxia examinations. He did not present neck resistance or negative meningeal irritation signs. The orientation to person, place, and time, calculation, and memory were normal in remission period.

During the attack period, routine blood tests showed that the patient's leukocyte count was 12.29 × 10^9^/L (above the normal range) and neutrophil percentage was 85.30% (above the normal range). Biochemical examination of blood plasma showed that fasting blood glucose was 6.48 mmol/L (above the normal range), glycosylated hemoglobin was 6.3%, and C‐reactive protein was 12.02 mg/L (above the normal range). Blood lipid profile revealed that cholesterol was 6.49 mmol/L (above the normal range), low density lipoprotein C was 5.24 mmol/L (above the normal range), and high density lipoprotein C was 1.06 mmol/L.

Tumor marker detection demonstrated that neuron‐specific enolase level was 25.40 ng/ml (above the normal range); electrolyte and liver and kidney functions were normal; thyroid‐stimulating hormone was 0.37 uIU/ml, free triiodothyronine was 2.18 pg/ml, free thyroxine was 10.13 pg/ml, thyroglobulin antibody was 3.21 IU/ml, and thyroid peroxidase antibody was 2.09 IU/ml.

Cerebrospinal fluid examination via lumbar puncture revealed that pressure was 120 mmH_2_O and cerebrospinal fluid was normal. Biochemical tests showed that glucose was 4.54 mmol/L, total protein was 0.71 g/L (above the normal range), and chloride was 119.0 mmol/L. Cytological examination of cerebrospinal fluid displayed 72% of lymphocytes and 28% of monocytes.

Electrophysiological test exhibited that sensory nerve conduction velocity of right median nerve (44.1 m/s), left common peroneal nerve (37.2 m/s), right common peroneal nerve (37.4 m/s), left tibial nerve (39.4 m/s), and right tibial nerve (37.3 m/s) was slow. At disease onset, electroencephalogram mainly demonstrated low amplitude α wave or α rhythm (8–10 Hz), and no special abnormal brain waves were observed.

**Figure 1:**
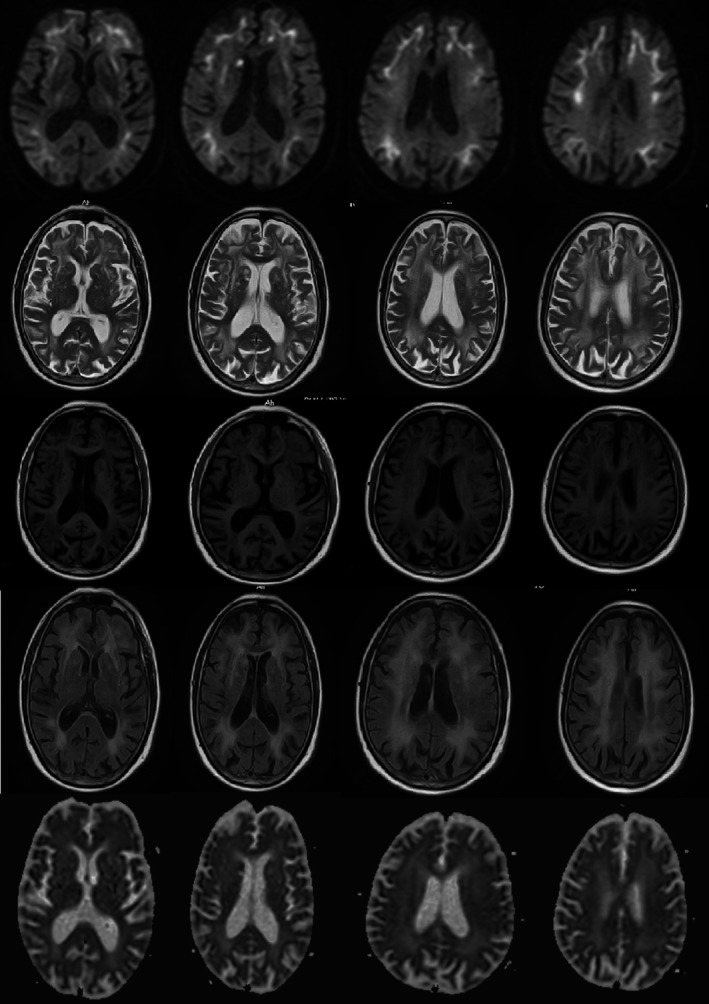
Diffusion‐weighted magnetic resonance imaging reveals symmetrically distributed strip‐shaped isointense signal in bilateral fronto‐occipital‐parietal cortical‐medullary junction, with nodular hyperintense signal in the right paraventricular white matter. The corresponding lesions in the cortical‐medullary junction showed isointense or slightly hyperintense signals on ADC sequence, and the corresponding lesions in the paraventricular region exhibited hypointense signal on ADC sequence. Multiple patchy hyperintense signals in bilateral paraventricular region, corona radiata, and semioval center were displayed on T2. The corresponding lesions showed hypointense signals on T1 sequence and hyperintense signals on Flair sequence

Cranial MRI revealed (Figure 1) multiple patchy long T1 and long T2 signals in bilateral paraventricular region, corona radiata, and semioval center. FLAIR sequences showed high signal intensity. Diffusion‐weighted imaging (DWI) revealed that strip‐shaped high‐intensity signals were symmetrically distributed in bilateral fronto‐occipital‐parietal cortical‐medullary junction. The corresponding lesions showed isointense or slightly hyperintense signals on ADC sequence, slightly hyperintense signal on T2 and FLAIR sequences, and isointense or slightly hypointense signals on T1 sequence.

**Figure 2:**
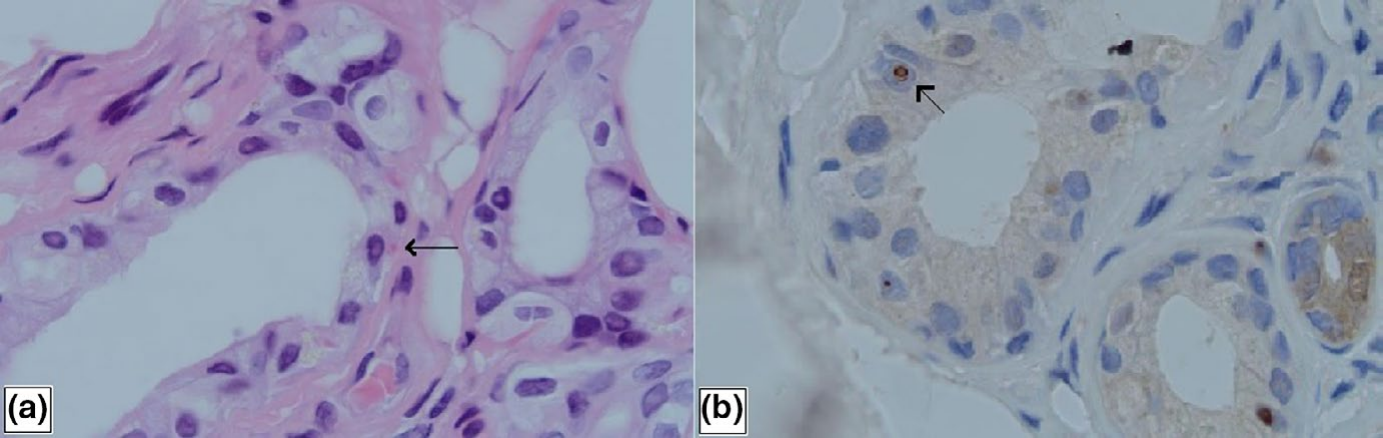
(a) Eosinophilic globular inclusion bodies (arrows) in the nucleus of sweat gland epithelial cells revealed by hematoxylin–eosin staining under the light microscope; (b) inclusion bodies (arrows) were positive for p62 measured by immunohistochemical staining

**Figure 3:**
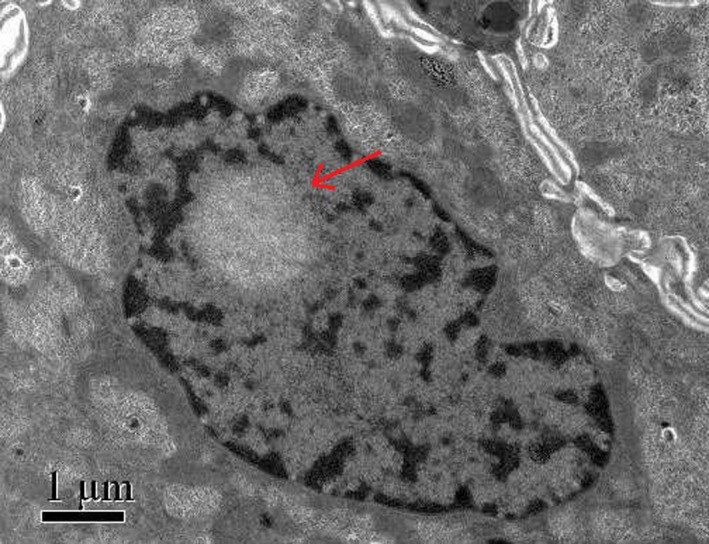
Spherical inclusion bodies composed of fibrous substances without membranes in the nucleus of sweat gland epithelial cells under the electron microscope

Skin biopsy was performed under light and electron microscopes. Hematoxylin–eosin staining demonstrated eosinophilic spherical inclusion bodies in nuclei of epithelial cells in the ductal parts of the sweat glands (Figure 2). Immunohistochemical staining showed that the inclusion bodies were positive for p62. Electron microscopy results demonstrated spherical inclusions without membranes in nuclei of epithelial cells in the ductal parts of the sweat glands (Figure 3), which was consistent with the typical pathological changes in skin of NIID.

The patient was given methylprednisolone pulse therapy (500 mg/daily) during hospitalization for three consecutive days. The condition was gradually relieved. Telephone follow‐up at 1 and 3 months after discharge showed that the condition of the patient was stable and there were no more disturbances of consciousness or psychosis. Occasionally, the patient experienced headache episodes, which could be tolerated and mitigated gradually. The patient could take care of himself and communicate with his family and neighbors.

## 3 DISCUSSION

The clinical manifestations of NIID can be classified into multiple groups of symptoms involved in the central nervous system, peripheral nerve, autonomic nerve, and other organs (Sone et al., 2016). Symptoms of central nervous system involvement include dementia, ataxia; conscious disturbance, abnormal behaviors, and encephalitis‐like performance. Moreover, a small number of patients presented with tremors, rigidity, and convulsions. Manifestations of peripheral nerve involvement include sensory disturbance and decreased muscle strength. Manifestations of autonomic nerve involvement include contracted pupil and bladder dysfunction. In the present study, the patient mainly presented with recurrent headache, abnormal mental behavior, and autonomic nervous system damage syndrome characterized by erectile dysfunction, dysuria, constipation, and sweating, and the persistent cognitive decline was not obvious, which further supports that acute encephalopathy combined with autonomic nervous system damage syndrome is the core clinical feature of NIID.

This patient also had characteristic imaging changes of NIID. That was, strip‐shaped high‐intensity signals were symmetrically distributed in bilateral fronto‐occipital‐parietal cortical‐medullary junction on DWI, mainly involving the frontal lobe, and spreading to parietal lobe, occipital lobe, and temporal lobe. The corresponding lesions showed isointense or slightly hyperintense signals on ADC sequence, multiple patchy long T1 and long T2 signals in bilateral paraventricular region, corona radiata, and semioval center, and hyperintense signal on FLAIR sequence. However, abnormal signals in the above regions were not displayed on the DWI sequence. This imaging change was considered to be associated with the presence of chronic persistent hypoperfusion‐induced cytogenic and angiogenic edema in the patient's brain (Araki et al., 2016). A previous study confirmed that symmetrically distributed strip‐shaped high‐intensity signals in bilateral fronto‐occipital‐parietal cortical‐medullary junction on DWI tended to extend backwards as the disease progressed and did not disappear over time (Sone et al., 2014). A recent study (Chen, Wu, et al., 2018; Chen, Li, Li, Zhu, Zhang, & Zhang, 2018) performed a 7‐year imaging follow‐up of NIID patients and found that occipital cortical lesions disappeared in the 5th year of follow‐up. The reason may be related to the decrease in local brain edema or the signal changes caused by neuronal loss and the proliferation of glial cells. Chen, Wu, et al. (2018) and Chen, Li, et al. (2018) reported that similar results, they found that cortical lesions disappeared over time, but the lesions persisted and gradually aggravated at the corticomedullary junction. The patient in this study had no abnormal signal in the cerebral cortex, but isolated high signal intensity on DWI was seen near the anterior crus of the right lateral ventricle. Different from the high signal intensity on DWI at the corticomedullary junction, the abnormal signal was a long T1 and long T2 signal, a low signal on the ADC sequence, and a high signal on the FLAIR sequence, and MRA suggested that the blood vessels were normal, which met the imaging findings of acute cerebral infarction. Moreover, this patient had risk factors for stroke, such as hypertension, smoking, and drinking. It was not excluded that the patient had hypertension‐related cerebral small vessel disease. NIID combined with lacunar infarction has not been reported yet. Previous studies (Chen, Wu, et al., 2018; Chen, Li, et al., 2018; Sone et al., 2016) confirmed that one of the characteristic imaging features of NIID was that the abnormal signal on DWI did not invade the deep white matter region. Nevertheless, neuronal intranuclear inclusions are extensively distributed in the body, so further studies are needed to investigate whether they invade vascular endothelial cells and cause lacunar infarction induced by cerebral small vessel disease. And further follow‐up observation is also needed to explore whether the isolated high signal intensity on DWI near the anterior crus of the right lateral ventricle disappears after a few months.

Electrophysiological test of this patient showed that the sensory and motor nerve conduction velocities in the upper and lower extremities were slow, and the amplitude was normal. It is considered that the peripheral nerve damage of this patient may still be associated with NIID. Although this patient had long‐term heavy drinking, a risk factor for peripheral neuropathy, but alcoholic peripheral neuropathy is an axonal, length‐dependent neuropathy, which cannot be explained by above electrophysiological results. Therefore, few studies have been reported on the electrophysiological characteristics of NIID. Hirose et al. (2018) proposed that the abnormalities of nerve conduction velocity and somatosensory evoked potential may be the diagnostic basis of NIID. Another study (Sone et al., 2016) verified that delay in conduction velocity or decrease in amplitude was found in motor and sensory nerves.

Neuronal intranuclear inclusion disease was diagnosed with autopsy, nerve biopsy, and rectal biopsy. Japanese scholars have promoted the widespread use of skin biopsy in the diagnosis of NIID and improved the clinical diagnosis rate of NIID. Sone et al. (2014) took three typical NIID patients as experimental group, one Alzheimer's disease patient and two Binswanger's disease patients as control group; the results showed that there were antiubiquitin‐positive intranuclear inclusions in adipocytes, fibroblasts, and sweat gland epithelial cells of the three NIID patients, but no intranuclear inclusions were found in the control group. Electron microscopy showed that intranuclear inclusions of the three patients were composed of filaments without membranes, which confirmed the specificity of skin biopsy in NIID diagnosis. In the present study, skin biopsy revealed eosinophilic spherical inclusions in the nucleus of sweat gland epithelial cells, and electron microscopy results confirmed the presence of the spherical intranuclear inclusions without membranes in the nucleus of sweat gland epithelial cells, but intranuclear inclusions were not found in adipocytes and fibroblasts, this may be related to the relatively short disease duration in our patient.

So far, the pathogenesis of NIID remains unclear. In 2019, Sone et al. (2019) discovered abnormal repeat amplification of GGC trinucleotides in NIID families, which provided a new idea for gene diagnosis of NIID. It is worth mentioning that the son of this patient died of suicide at the age of 20 years. Whether his personality changes before suicide were also caused by genetic anticipation in NIID still has a certain possibility. But in the present study, the patient refused to have genetic test.

In summary, NIID has a low incidence, diverse clinical manifestations, and difficulty in early diagnosis. Our case verified that the disease has typical clinical manifestations and suggestive MRI features. The use of skin biopsy with minimal trauma can promptly diagnose NIID and improve the awareness of the clinicians.

## CONFLICT OF INTEREST

The authors have declared no conflicts of interest.

## Data Availability

The data that support the findings of this study are available from the corresponding author upon reasonable request.
